# Supplementary figures and images for: Human cell-dependent, directional, time-dependent changes in the mono- and oligonucleotide compositions of SARS-CoV-2 genomes
Source: BMC Microbiol. 2021 Mar 23;21:89. doi: 10.1186/s12866-021-02158-6 (PMC7987243; doi:10.1186/s12866-021-02158-6)

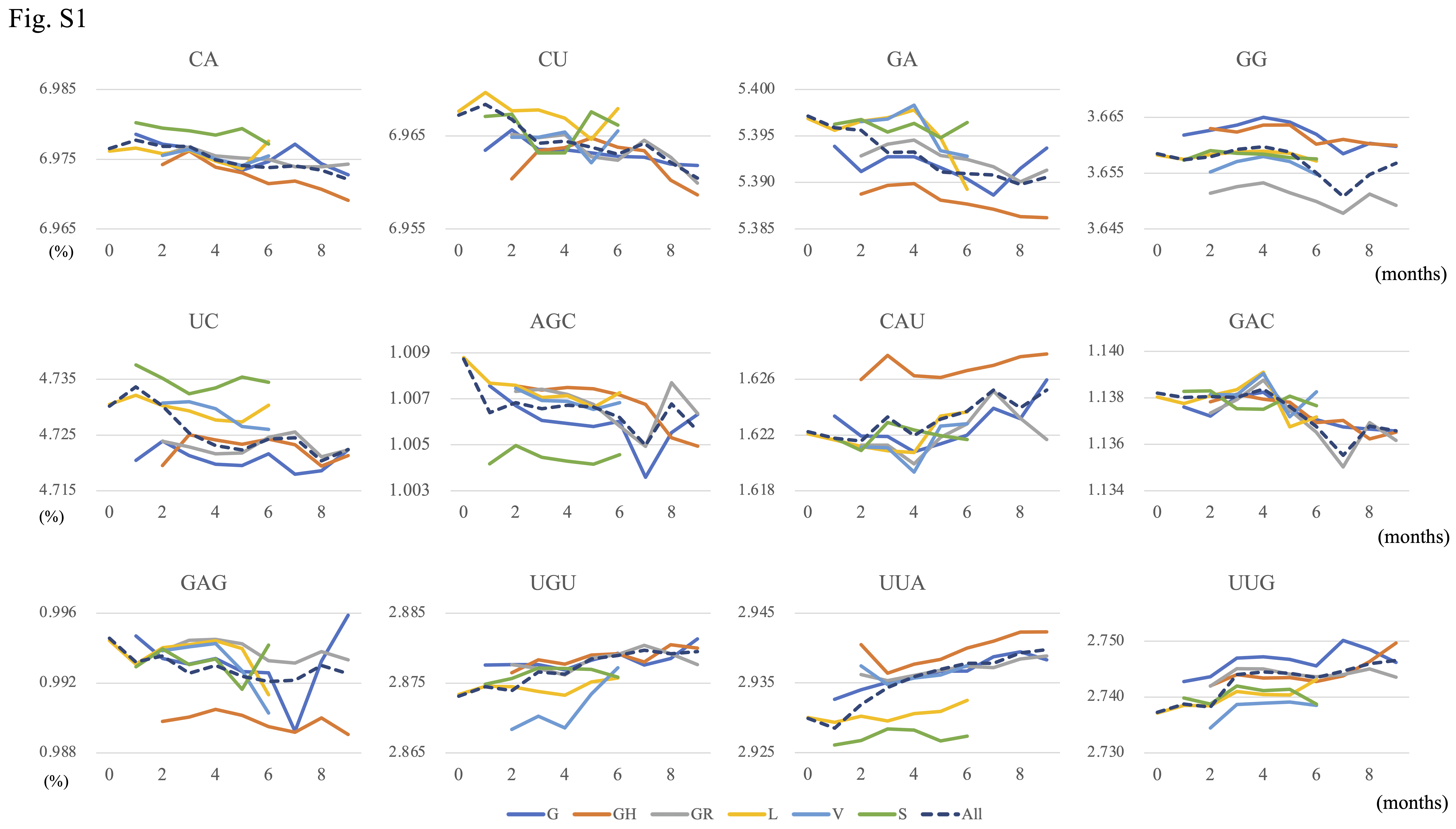

Supplement: Supplementary file 1 — Additional file 1: Fig. S1. Average di- and trinucleotide compositions (A and B) of for SARS-CoV-2 strains collected in each elapsed month. Fig. S2. Oligonucleotide compositions of human and bat coronavirus sequences. Fig. S3. Differences in oligonucleotide composition between SARS-CoV-2 and human-CoV. [file 12866_2021_2158_MOESM1_ESM.zip › Additional_file_1_Fig_S1.png]

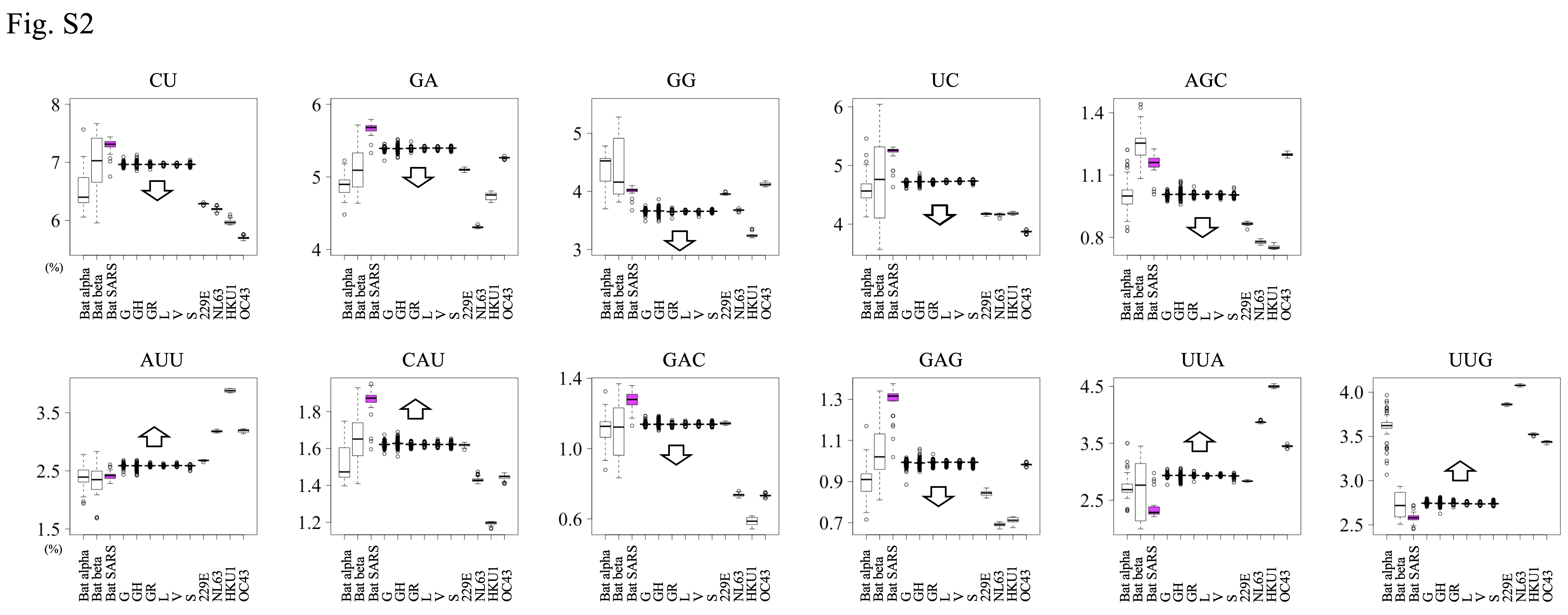

Supplement: Supplementary file 1 — Additional file 1: Fig. S1. Average di- and trinucleotide compositions (A and B) of for SARS-CoV-2 strains collected in each elapsed month. Fig. S2. Oligonucleotide compositions of human and bat coronavirus sequences. Fig. S3. Differences in oligonucleotide composition between SARS-CoV-2 and human-CoV. [file 12866_2021_2158_MOESM1_ESM.zip › Additional_file_1_Fig_S2.png]

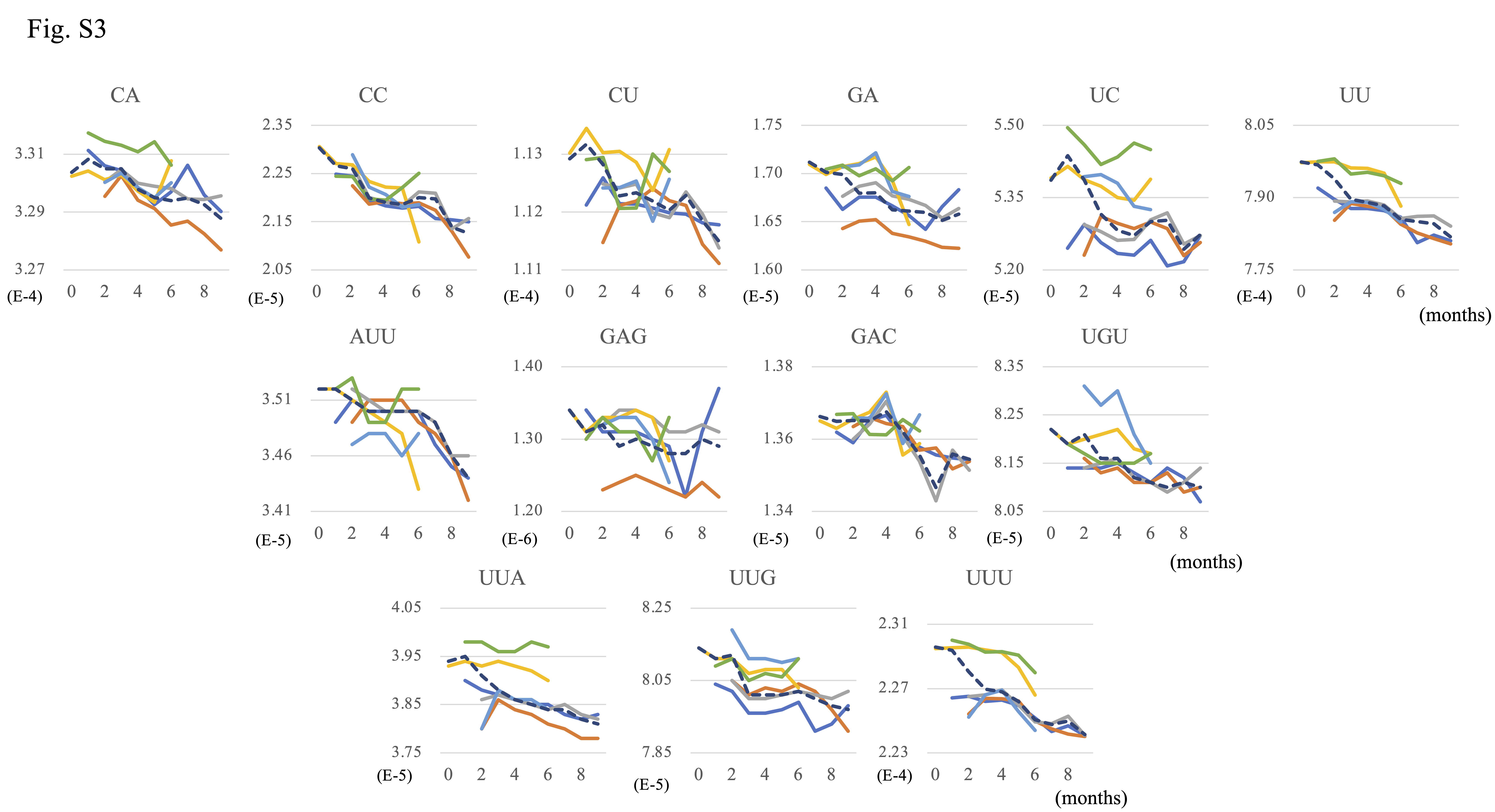

Supplement: Supplementary file 1 — Additional file 1: Fig. S1. Average di- and trinucleotide compositions (A and B) of for SARS-CoV-2 strains collected in each elapsed month. Fig. S2. Oligonucleotide compositions of human and bat coronavirus sequences. Fig. S3. Differences in oligonucleotide composition between SARS-CoV-2 and human-CoV. [file 12866_2021_2158_MOESM1_ESM.zip › Additional_file_1_Fig_S3.png]
